# The Value of a Virtual Assistant to Improve Engagement in Computerized Cognitive Training at Home: Exploratory Study

**DOI:** 10.2196/48129

**Published:** 2024-06-20

**Authors:** Isabella Zsoldos, Eléonore Trân, Hippolyte Fournier, Franck Tarpin-Bernard, Joan Fruitet, Mélodie Fouillen, Gérard Bailly, Frédéric Elisei, Béatrice Bouchot, Patrick Constant, Fabien Ringeval, Olivier Koenig, Hanna Chainay

**Affiliations:** 1 Laboratoire d’Étude des Mécanismes Cognitifs Université Lumière Lyon 2 Lyon France; 2 SBT Humans Matter Lyon France; 3 GIPSA-Lab Université Grenoble Alpes Grenoble France; 4 Atos Echirolles France; 5 Pertimm Asnières-sur-Seine France; 6 Laboratoire d’Informatique de Grenoble Université Grenoble Alpes Grenoble France

**Keywords:** cognitive training, cognitive decline, cognitive disorders, mild cognitive impairment, Alzheimer disease, digital therapies, virtual health assistant, conversational agent, artificial intelligence, social interaction, THERADIA

## Abstract

**Background:**

Impaired cognitive function is observed in many pathologies, including neurodegenerative diseases such as Alzheimer disease. At present, the pharmaceutical treatments available to counter cognitive decline have only modest effects, with significant side effects. A nonpharmacological treatment that has received considerable attention is computerized cognitive training (CCT), which aims to maintain or improve cognitive functioning through repeated practice in standardized exercises. CCT allows for more regular and thorough training of cognitive functions directly at home, which represents a significant opportunity to prevent and fight cognitive decline. However, the presence of assistance during training seems to be an important parameter to improve patients’ motivation and adherence to treatment. To compensate for the absence of a therapist during at-home CCT, a relevant option could be to include a virtual assistant to accompany patients throughout their training.

**Objective:**

The objective of this exploratory study was to evaluate the interest of including a virtual assistant to accompany patients during CCT. We investigated the relationship between various individual factors (eg, age, psycho-affective functioning, personality, personal motivations, and cognitive skills) and the appreciation and usefulness of a virtual assistant during CCT. This study is part of the THERADIA (Thérapies Digitales Augmentées par l’Intelligence Artificielle) project, which aims to develop an empathetic virtual assistant.

**Methods:**

A total of 104 participants were recruited, including 52 (50%) young adults (mean age 21.2, range 18 to 27, SD 2.9 years) and 52 (50%) older adults (mean age 67.9, range 60 to 79, SD 5.1 years). All participants were invited to the laboratory to answer several questionnaires and perform 1 CCT session, which consisted of 4 cognitive exercises supervised by a virtual assistant animated by a human pilot via the Wizard of Oz method. The participants evaluated the virtual assistant and CCT at the end of the session.

**Results:**

Analyses were performed using the Bayesian framework. The results suggest that the virtual assistant was appreciated and perceived as useful during CCT in both age groups. However, older adults rated the assistant and CCT more positively overall than young adults. Certain characteristics of users, especially their current affective state (ie, arousal, intrinsic relevance, goal conduciveness, and anxiety state), appeared to be related to their evaluation of the session.

**Conclusions:**

This study provides, for the first time, insight into how young and older adults perceive a virtual assistant during CCT. The results suggest that such an assistant could have a beneficial influence on users’ motivation, provided that it can handle different situations, particularly their emotional state. The next step of our project will be to evaluate our device with patients experiencing mild cognitive impairment and to test its effectiveness in long-term cognitive training.

## Introduction

### Background

Impaired cognitive function is observed in many pathologies, including neurodegenerative diseases, neurodevelopmental disorders, and certain psychiatric disorders (eg, depression and schizophrenia). The most prevalent cause of cognitive decline is dementia, for which aging is the main risk factor. According to the World Health Organization [1], 55 million people are currently affected by dementia worldwide, and this number could increase to 139 million by 2050. Dementia is a chronic and progressive syndrome characterized by an impairment of cognitive functions such as memory, reasoning, language, and executive functions. At advanced stages, it severely affects autonomy and quality of life, making it a major public health concern. Alzheimer disease is the most common cause of dementia (60% to 70% of the cases), but there are other potential causes (eg, vascular, Lewy bodies, and Parkinson disease) [2].

At present, there is no effective pharmacological treatment for the symptoms of Alzheimer disease and dementia. Cholinesterase inhibitors and memantine offer only modest and short-term cognitive benefits, with substantial side effects [3-6]. Because of the controversial effectiveness of the existing pharmacological treatments, there has been a strong research interest in developing nonpharmacological treatments that are safe, noninvasive, and with few side effects. The main objective of these treatments is to preserve the quality of life and autonomy of patients for as long as possible. They encompass a wide range of techniques, such as cognitive intervention (including cognitive stimulation, cognitive training, and cognitive rehabilitation), motor rehabilitation, psychotherapy, occupational therapy, and assistive technologies [7].

A nonpharmacological treatment that has received considerable attention is computerized cognitive training (CCT), which aims to maintain or improve cognitive functioning through repeated practice in standardized exercises [8]. CCT targets one or more cognitive domains (eg, memory and attention) and adapts exercise difficulty to individual performance. These therapies have many advantages: they are safe and relatively inexpensive and allow patients to train their cognitive functions on a more regular basis by conducting sessions at home, eliminating the need to travel to the therapist’s office or hospital. Regarding effectiveness, meta-analyses of randomized controlled trials reported significant but moderate effects of CCT in healthy older adults [9], in patients with Parkinson disease [10,11] and mild cognitive impairment (MCI) [8,12]. MCI refers to the transitional state between normal aging and dementia, which is characterized by a greater cognitive decline than what is considered normal for a given individual (based on age and education), but not significant enough to affect autonomy in daily life [13]. Individuals with MCI have a high probability of progressing to dementia, but this is not systematic [2,13]. Once dementia is diagnosed, CCT appears to become ineffective in countering cognitive decline [8].

There is currently no consensus on the best time to start cognitive training to prevent cognitive impairment in older individuals. The available data suggest an improvement in cognitive functioning in healthy older adults who receive cognitive training, whereas the results are more mixed in those already experiencing cognitive impairment [9,14-17]. In addition, there is still insufficient evidence to support a preventive effect of cognitive training on the onset of cognitive disorders or dementia in the long term [14,16]. It is however reasonable to hypothesize that the earlier cognitive training begins, the more beneficial the effects on cognitive functioning could be, in line with the cognitive reserve theory [18,19]. Further research is needed to test whether CCT is a promising tool for the prevention of cognitive decline in healthy older adults and an effective treatment for patients with MCI.

In general, the effectiveness of cognitive training in preserving or improving cognitive function is still debated in the literature [14,20,21]. Methodological issues (eg, unclear randomization methods and inadequate sample sizes) have often been put forward as an explanation for the moderate effects of CCT and the lack of a strong consensus across studies [7,15,21,22]. Nevertheless, other important factors related to the format of training programs and to individual differences are likely to impact CCT effectiveness. A meta-analysis in particular showed that unsupervised at-home CCT is less beneficial for cognitive function than group-based CCT [9]. The main difference is that group-based CCT involves social interactions and the presence of a therapist who ensures adherence, treatment fidelity, compliance, and computer assistance. The therapist and social dimension are absent when patients perform CCT at home, which may decrease the motivation to complete or succeed in the exercises. Motivation plays a key role in CCT success, as well as other individual factors such as preexisting ability and the need for cognition (ie, how much one enjoys cognitively challenging tasks) [23].

CCT allows for more regular and thorough training of cognitive functions directly at home, which represents a significant opportunity to fight cognitive decline. However, the design of CCT needs to be reconsidered to address parameters that may reduce therapy effectiveness. Various individual factors can represent limitations for performing CCT at home, such as personal motivations and familiarity with computers, as well as psychological factors such as anxiety level, mood, or personality. From the abovementioned evidence, the presence of assistance during training seems to be an important parameter to improve patients’ motivation, adherence to treatment, and thus benefits on cognition. To compensate for the absence of a therapist during at-home CCT, a relevant option could be to include a virtual assistant to accompany patients throughout their training.

The addition of a virtual assistant in CCT seems to be particularly relevant for older adults with or without cognitive impairment, who are the main targets of cognitive training. To our knowledge, there are currently no published studies assessing the benefits of a virtual assistant to accompany individuals during CCT. However, outside cognitive training, some studies suggest that older adults do appreciate assistive technologies such as virtual home assistants (eg, Amazon Echo Alexa and Google Home) [24-26], conversational agents [27-29], and social robots [30] to help them with daily activities. Older adults find virtual home assistants useful for setting reminders, searching for information in real time, and entertainment [24-26,29]. They appreciate the interaction with the assistant and its companionship [26]. As for applications dedicated to care and health, the few studies available suggest a good perception by older adults of the support provided by virtual companions [27,31]. Older adults seem to prefer embodied to nonembodied virtual assistants, particularly assistants with humanoid rather than zoomorphic or machine-like features [32-34], female rather than male assistants [34,35], and assistants that are not too realistic [33]. However, it was observed that movement realism had a more positive impact on user satisfaction and interaction quality than the appearance of the assistant (eg, graphics and texture quality) [36]. A recent literature review suggested that patients with dementia enjoy interacting with embodied conversational agents, although data on this topic are still scarce [37]. Regarding social robots, there is some evidence that robot-assisted cognitive training can improve memory and executive function in older adults [38]. Social robots also have a positive influence on well-being [30]. However, such robots are currently too expensive to be implemented at home, so patients must travel to centers to benefit from their assistance during training. A virtual assistant may represent a less expensive and easier solution to implement in the patient’s home.

In addition to assisting patients in their cognitive training exercises, a virtual assistant could be capable of less formal social interactions (eg, small talk) and provide cognitive stimulation. Cognitive stimulation is a type of cognitive intervention that consists of various activities aimed at enhancing an individual’s overall cognitive and social functioning [7]. It has been shown to improve general cognitive functioning in patients with mild-to-moderate dementia [7]. The combination of cognitive training, cognitive stimulation, and social interactions provided by a virtual assistant could thus be beneficial for patients’ motivation and long-term adherence to CCT. Moreover, some data suggest that individuals might build stronger therapeutic alliances with a conversational agent than with a human caregiver in certain contexts (eg, major depression) [39]. Many older adults with cognitive disorders are embarrassed by their condition and may be more willing to interact with a virtual, anonymous device for help or advice than with humans [40].

Finally, certain design parameters are particularly important to consider when developing an effective virtual assistant to accompany older adults, with or without cognitive impairment, during CCT at home. In addition to the appearance and animation quality discussed earlier, talking virtual assistants rather than silent ones appear to improve the engagement of older adults with low computer literacy [35], which patients with cognitive disorders are likely to be. More generally, the simultaneous presence of visual and auditory modalities when interacting with the assistant could improve the acceptance and user experience of older adults [41]. The virtual assistant must be able to provide adequate emotional support during the session, encouraging and rewarding participants for their efforts, to increase adherence [40]. In this respect, the development of an emotional artificial intelligence that would enable the assistant to detect and automatically adapt to the user’s affective states would be particularly useful [42]. To provide a safe environment for patients with cognitive disorders, it is also necessary that the assistant’s speech and its interactions with the user are scripted in such a way as to provide a stable and rather predictable framework [40].

### Objectives

In the light of these observations, we started the THERADIA (Thérapies Digitales Augmentées par l’Intelligence Artificielle) project in 2020 [42]. This 5-year project aims to develop an empathetic virtual assistant that can accompany users during at-home CCT. The first version of our CCT software will be targeted at older adults with or without cognitive disorders, with the aim of maintaining, or even improving, cognitive functioning. To successfully complete this project, it was first necessary to better understand the factors that may contribute to the effectiveness of such a device. As discussed earlier, users’ characteristics play an important role in the adherence to CCT programs. Therefore, the objective of this study was to investigate the relationship between various individual factors (eg, age, psycho-affective functioning, personality, personal motivations, and cognitive skills) and the appreciation and usefulness of a virtual assistant during CCT. To do so, young and older adults were invited to the laboratory to answer several questionnaires and perform 1 CCT session hosted by a virtual assistant, animated by a human pilot via the “Wizard of Oz” method. This exploratory study thus presents 1 stage of the development of the future virtual assistant that will be proposed by the THERADIA consortium.

## Methods

### Participants

Although older adults are the first target for our future cognitive training software with virtual assistance, young adults can also experience cognitive disorders in certain situations (eg, after a stroke or in certain psychiatric conditions). As computer skills may vary with age, older adults may not have the same abilities or needs as young adults when performing CCT at home. Therefore, we included both young and older adults in our study to explore age-related differences in the evaluation of our device, with the goal of potentially adapting it to a younger population in the future.

To determine the sample size, we relied on the available literature whose objectives were closest to our own, that is, to investigate the appreciation and preferences of older adults regarding virtual assistants in general [24,26-29,31-36]. Most of these studies used qualitative research methods (focus groups or interviews) involving small experimental groups of 5 to 24 older adults [24,26,28,29,32,34,36]. Studies using quantitative research methods included 20 to 46 older adults per experimental group, with 46 participants being more common [27,31,33,35]. On the basis of studies using quantitative research methods, more similar to our study design and analysis plan, we decided to slightly increase the number of participants usually included to 52 per age group to improve power.

Therefore, a total of 104 healthy participants were recruited between April 2021 and September 2022, including 52 (50%) young and 52 (50%) older adults. The key characteristics of the participants are summarized in Table 1. Inclusion criteria were to be aged between 18 and 30 years for young adults and >60 years for older adults. All participants were French speakers; had normal or corrected-to-normal vision and hearing; and were free from known psychiatric conditions, neurological disorders, and neurodegenerative diseases. They also had to confirm that they were not undergoing any treatment (eg, medication, therapy, or inclusion in another study) likely to affect memory or movement. Older participants presenting altered cognitive functions (a score <25 at the Mini-Mental State Examination [MMSE] [43]) were excluded from the analysis.

**Table 1:** Description of participants by age group (N=104).

Group		Young adults (n=52)	Older adults (n=52)
**Sex, n (%)**			
	Female	35 (67)	40 (77)
	Male	17 (33)	12 (23)
Age (y), mean (SD; range)		21.17 (2.90; 18-27)	67.92 (5.14; 60-79)
**Highest diploma obtained, n (%)**			
	CAP^a,b^	1 (2)	6 (12)
	Baccalaureate^c^	33 (63)	8 (15)
	Bachelor’s degree	15 (29)	16 (31)
	Master’s degree	3 (6)	19 (37)
	PhD^d^	0 (0)	3 (6)
**Occupation, n (%)**			
	Student	43 (83)	0 (0)
	Retired	0 (0)	47 (90)
	Employee	4 (8)	3 (6)
	Executive or manager	2 (4)	1 (2)
	Worker or laborer	1 (2)	0 (0)
	Company director	0 (0)	1 (2)
	Unemployed	2 (4)	0 (0)

^a^CAP: Certificat d’Aptitude Professionnelle.

^b^Equivalent to the NVQ (National Vocational Qualification) in the United Kingdom.

^c^Equivalent to A-levels in the United Kingdom and high-school diploma in the United States.

^d^PhD: Doctor of Philosophy.

The young adults were recruited on the campus of the Université Lumière Lyon 2 via mail announcements as well as diffusion on social networks such as Facebook (Meta platforms, Inc). For the older adults, 2 advertisements were published in regional newspapers: Le Progrès and Le Dauphiné Libéré. A campaign to recruit older adults was also carried out by advertising to people enrolled in a teaching program open to individuals of all ages (“University of All Ages”) attached to the Université Lumière Lyon 2.

### Ethical Considerations

This study was approved by the Ethics Committee of the Université Grenoble Alpes (CERGA-Avis-2021-1). All participants provided written informed consent before starting the experiment. At the end of the experiment, each participant received a €20 (US $21) gift card as a reward.

### Evaluation of Individual Characteristics

#### Overview

Several characteristics of the young and older participants were assessed along four dimensions: (1) psycho-affective functioning, (2) personality, (3) personal motivations, and (4) personal habits. We also assessed the cognitive functions of the older adults to ensure that they were not experiencing cognitive decline and to test the relationship between cognitive functioning and the evaluation of the virtual assistant. These dimensions of interest were selected to provide a global view of the participants’ psychological and cognitive functioning, including stable parameters (eg, personality traits, motivational factors, and habits) and more fluctuating parameters (eg, current emotional state and state anxiety). Each dimension was studied using specific questionnaires in paper form, which are summarized in Table 2 and described in detail subsequently.

**Table 2:** Summary of the questionnaires used to assess various psychological and cognitive characteristics of the participants by dimension and subdimensions investigated.

Dimension, subdimensions, and questionnaires				Scores calculated
**Psycho-affective functioning**				
	**Global affective experience**			
		Modified PANAS^a^ [44]	Positive affect score Negative affect score	
	**Current affective state**			
		Modified SAM^b^ [45]	Intrinsic relevance Controllability Arousal Novelty Goal conduciveness	
		BMIS^c^ [46]	Pleasant-unpleasant Arousal-calm	
	**Anxiety**			
		STAI-Y^d^ (French version) [47]	State anxiety Trait anxiety	
**Personality**				
	**Extraversion, agreeableness, conscientiousness, emotional stability, and openness**			
		TIPI^e^ [48]	Extraversion Agreeableness Conscientiousness Emotional stability Openness	
**Personal motivations**				
	**Intrinsic motivation, extrinsic motivation, and amotivation**			
		GMS^f^-28 [49]	Knowledge Accomplishment Stimulation Introjected motivation Identified motivation External motivation Amotivation	
**Personal habits and cognitive abilities**				
	**Cognitive abilities and habits**			
		Cognitive abilities and habits (homemade questionnaire)	Familiarity with computers Familiarity with cognitive exercises Familiarity with cognitive training Memory difficulty Attentional difficulty Playing a musical instrument Playing board games Playing games such as chess or crossword puzzles Sports and exercise Meditation and relaxation	
**Cognitive functioning (older adults only)**				
	**Global cognitive function**			
		MMSE^g^ [43]	Total score	
	**Executive functions**			
		TMT^h^ [50]	Execution time (in seconds) Number of errors	
		FAB^i^ [51]	Total score	
	**Memory**			
		5WT^j^ [52]	Total score	

^a^PANAS: Positive and Negative Affect Schedule.

^b^SAM: Self-Assessment Manikin.

^c^BMIS: Brief Mood Introspection Scale.

^d^STAI-Y: State-Trait Anxiety Inventory.

^e^TIPI: Ten-Item Personality Inventory.

^f^GMS: Global Motivation Scale.

^g^MMSE: Mini-Mental State Examination.

^h^TMT: Trail Making Test.

^i^FAB: Frontal Assessment Battery.

^j^5WT: 5 Words Test.

#### Psycho-Affective Functioning

We studied the psycho-affective functioning of the participants according to 3 aspects: general affective functioning in everyday life, affective state at the time of the session (emotions and mood), and anxiety level.

On the basis of the Positive and Negative Affect Schedule [44], we constructed a 39-item scale to measure the participants’ general affective experience. The items were words describing positive and negative affects, and the participants were asked to indicate how frequently they experienced each one of these affects during the last 6 months using a 7-point scale ranging from 1 (“never”) to 7 (“several times a day”). A positive affect score and a negative affect score were calculated separately.

A modified Self-Assessment Manikin [45] was used to assess the current affective state of the participants at the time of the session. They were instructed to rate their affective state toward the present situation with a 9-point scale along 5 dimensions: intrinsic relevance, controllability, arousal, novelty, and goal conduciveness. Intrinsic relevance refers to the current level of pleasure felt and was rated from 1 (“unpleasant”) to 9 (“pleasant”). Controllability reflects the feeling of control over the situation, ranging from 1 (“uncontrollable”) to 9 (“controlled”). Arousal refers to the physiological and psychological state of being awake and alert and was rated from 1 (“sleep”) to 9 (“excitation”). As some authors have pointed out that 3 dimensions are not sufficient to capture the current affective state of individuals [53], we included 2 supplementary dimensions that are considered essential in emotional episodes according to appraisal theories of emotion [54], namely, novelty and goal conduciveness. Novelty refers to the feeling of novelty of the current situation and was rated on a scale from 1 (“predictable”) to 9 (“surprising”). Goal conduciveness refers to the consistency of the situation with current achievement concerns and was rated on a scale from 1 (“obstructive”) to 9 (“conducive”).

The second scale used to assess the current affective state of participants was the Brief Mood Introspection Scale [46] including 16 mood adjectives. Participants were asked to rate the extent to which each adjective described their current mood on a 4-point scale ranging from XX (“definitely do not feel”) to VV (“definitely feel”). A total of 2 mood scores were calculated on the following scales: pleasant-unpleasant (valence dimension) and arousal-calm (arousal dimension). For each scale, the higher the score, the more the current state of the participant tended toward the first cited component (such as “pleasant” for the pleasant-unpleasant scale).

The French version of the State-Trait Anxiety Inventory [47] was used to evaluate participants’ anxiety. This questionnaire is divided into 2 subscales, one measuring the current state of anxiety (S-Anxiety) and the other measuring the anxiety trait in general (T-Anxiety). The S-Anxiety scale consists of 20 items describing current statements (eg, “I feel safe” and “I feel blue”) that participants were asked to rate from 1 (“not at all”) to 4 (“very much so”) to indicate how they feel “right now.” The T-Anxiety scale contains 20 items of statements that participants feel in general. Participants were asked to rate from 1 (“almost never”) to 4 (“almost always”) the extent to which each of the statements corresponded to them. Therefore, the total score from both scales varies from 20 to 80. The higher the score, the higher the level of anxiety.

#### Personality

The Ten-Item Personality Inventory [48] was used to measure the personality traits of the participants: extraversion, agreeableness, conscientiousness, emotional stability, and openness to experience. Participants were asked to rate how well a pair of personality traits matched them by choosing on a 7-point scale from 1 (“disagree strongly”) to 7 (“agree strongly”). An average of the 2 items by dimension was calculated. The higher the score, the more the participant tended toward the dimension trait.

#### Personal Motivations

The Global Motivation Scale-28 [49] was used to assess the personal motivations of our participants. It includes 28 items, each of which describes a possible reason that drives individuals to act in their lives (eg, “In general, I do things because I like making interesting discoveries”). The participants were asked to indicate the extent to which each of the statements corresponded to the reasons why they do different things in general on a 7-point scale ranging from 1 (“does not correspond accordingly”) to 7 (“corresponds completely”). A total of 7 scores were calculated that reflect different motivations: intrinsic motivation (toward knowledge, accomplishment, and stimulation), extrinsic motivation (identified, introjected, and external regulation), and amotivation. The higher the score, the more the source of motivation influenced the participant’s behavior.

#### Personal Habits and Cognitive Abilities

To measure personal habits and cognitive abilities, we created a 10-item questionnaire divided into 3 parts. In the first part, participants rated their familiarity with computers, cognitive exercises, and cognitive training from 1 (“very weak”) to 5 (“very strong”). In the second part, participants rated their attentional and memory difficulty from 1 (“a lot of difficulties”) to 5 (“very few difficulties”). In the last part, participants rated how often they practice different activities from 1 (“never”) to 5 (“very often”): playing musical instruments, playing board games, playing chess, solving crossword puzzles, playing sports, and meditation.

#### Cognitive Functioning (Older Adults Only)

We used 4 questionnaires to assess cognitive functions in older adults: the MMSE [43], Trail Making Test (TMT) [50], Frontal Assessment Battery (FAB) [51], and 5 Words Test [52]. All these tests are widely used to detect cognitive decline associated with dementia syndromes.

The MMSE was administered to investigate global cognitive functioning. It consists of 30 items measuring different cognitive abilities in a few minutes (eg, attention, memory, language, and calculation) and provides a total score out of 30 that gives a global view of cognitive functioning (the higher the score, the better the cognitive abilities). A score of 23 out of 30 is the generally accepted cutoff indicating the presence of cognitive impairment.

The TMT and FAB were used to assess executive function. Successful completion of the TMT requires several cognitive skills, such as visual scanning and mental flexibility. The TMT is divided into 2 parts. In Part A, measuring the speed of processing, the participants had to connect numbers in ascending order (from 1 to 25) as quickly as possible and without error, and in Part B, measuring mental flexibility, the participants had to connect numbers and letters in alternating and increasing order (ie, 1, A, 2, B, and so on). Slower execution time and a higher number of errors, compared to the norms of the tested population, indicate a decline in executive functions.

The FAB was used to assess frontal lobe function and screen for dysexecutive disorders through 6 subtests that examine different cognitive functions: abstract reasoning, mental flexibility, motor programming, interference sensitivity, inhibitory control, and environmental autonomy. A total score <16 out of 18 indicates the possibility of an executive function disorder.

Finally, we used the 5 Words Test to examine episodic memory. This test consists of evaluating the memorization of a short list of words in 4 steps: a learning phase, an immediate free and cued recall, an interfering task, and then a delayed free and cued recall. A total score should normally equal 10.

### Evaluation of the Virtual Assistant

A specific questionnaire, administered in paper form, was created for the evaluation of the virtual assistant. It contained 10 items investigating the participants’ opinion on the virtual assistant and its impact on cognitive training across main dimensions: (1) overall appreciation of the assistant, (2) impact of the assistance on the comprehension of the exercises, (3) impact on motivation, and (4) personality of the assistant. Although the assistant was animated by a human pilot, an evaluation of the assistant’s personality was included to explore some design features that users might be sensitive to and that might influence their motivation to interact with the assistant and complete the cognitive exercises (ie, sense of humor and familiarity). The participants responded to each item using visual analog scales ranging from 0 to 10 cm, which were then rated in millimeters to calculate 7 scores exploring the dimensions of interest (Table 3). Of the 7 scores, 3 (appreciation, comprehension, and engagement) were calculated as the mean of 2 items.

**Table 3:** Synthesis of the items used and the scores calculated to evaluate the virtual assistant by dimension.

Dimensions examined, scores calculated, and items				Response (visual analog scales)
**Overall appreciation of the virtual assistant**				
	“**Appreciation”**			
		In general, did you find that the virtual assistant accompanied you well during the cognitive training session?	From “not at all” to “absolutely”	
		If you had to do several cognitive training sessions per week at home, would you like to be accompanied by a virtual assistant like this one?	From “not at all” to “absolutely”	
**Impact of virtual assistance on comprehension**				
	“**Comprehension”**			
		Did you always understand what you were supposed to do in the exercises?	From “not at all” to “absolutely”	
		Were the instructions and tips given by the virtual assistant useful for you to do your exercises?	From “not at all” to “absolutely”	
**Impact of virtual assistance on motivation**				
	“**Engagement”**			
		How would you rate your level of engagement in the exercises that you have done?	From “very weak” to “very strong”	
		Did you feel able to perform the exercises?	From “never” to “Always”	
	“**Desire to give up”**			
		Did you ever feel like giving up the session?	From “never” to “all the time”	
	“**Fatigue level”**			
		After this session, how would you rate your level of fatigue?	From “not at all tired” to “extremely tired”	
**Personality of the virtual assistant**				
	“**Familiarity”**			
		Regarding the behavior of the virtual assistant, would you prefer it to be more or less familiar?	From “less familiar” to “more familiar”	
	“**Sense of humor”**			
		Regarding the virtual assistant’s sense of humor, would you like it to be more or less humorous?	From “less humor” to “more humor”	

### CCT and Wizard of Oz Method

The participants performed the CCT on a Dell (Dell Inc) computer with a diagonal monitor width of 24 inches. The CCT consisted of 4 exercises that were selected from the HappyNeuronPro cognitive training program designed by Humans Matter (Lyon, France), a company providing services for health and paramedical professionals such as speech therapists and neuropsychologists. The selected exercises engaged different cognitive functions such as memory, language, attention, and planification.

**Figure 1:**
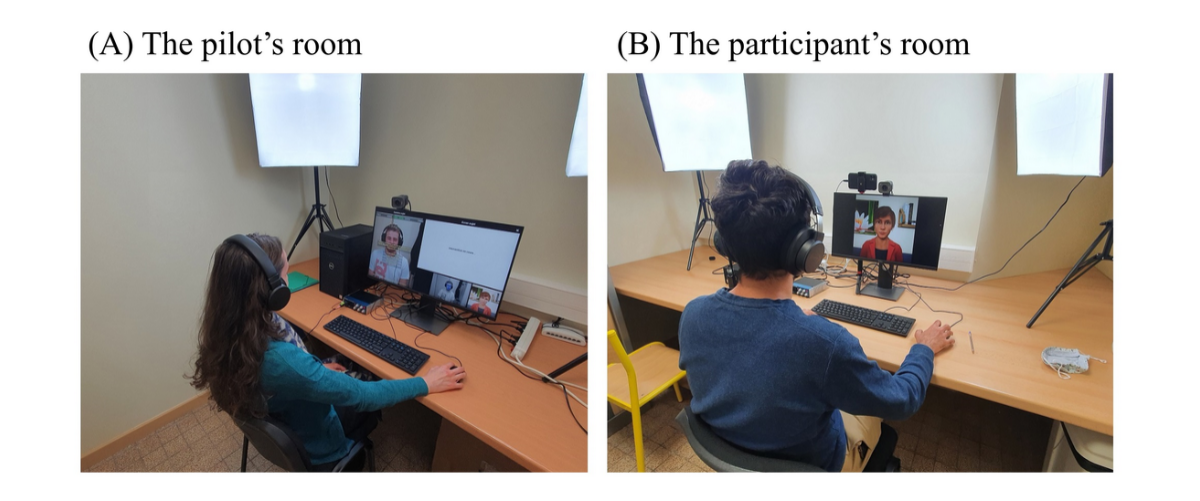
Wizard of Oz device.

During the CCT session, the participant was guided by a virtual assistant and could interact with her. The CCT was conducted via the software developed for this purpose by the Atos company (Echirolles, France), which allowed alternating appearances of the virtual assistant and the exercises. In reality, the virtual assistant was animated by a human pilot via the so-called Wizard of Oz method, that is, the pilot was in another room, and the participant was not informed of her existence (refer to Figure 1 for pictures of the Wizard of Oz device). All sessions were led by the same pilot.

The pilot sat in front of a Dell computer identical to that of the participant. With a high-quality camera, we used the facial motion capture solution proposed by the Dynamixyz company (Rennes, France) to drive, in real time, the head and face movements of a 3D avatar from those of the human pilot via video analysis. A humanlike appearance was chosen for the avatar, in line with the literature suggesting that older adults prefer to interact with humanoid virtual assistants [32-34] especially with feminine features [34,35]. The avatar represented a woman in her thirties, with fair skin and short brown hair, wearing a red jacket. The avatar was displayed from the front, with the head, shoulders, and upper arms visible. She appeared on a 3D background simulating the office of a health professional, similar to those of neuropsychologists or speech therapists who usually perform cognitive remediation. The image of the avatar was transmitted in real time on the participant’s screen via the software developed by Atos. Conversely, a webcam also transmitted the participant’s face in real time to the pilot’s screen so that the pilot could follow the participant’s gaze and movements during the discussions to make them more natural. The videos of the pilot and participant were recorded for later use in the development of the empathic virtual assistant proposed by the THERADIA consortium [42]. The pilot and participant communicated via headsets with integrated microphones, and no audio processing was performed to alter the pilot’s voice.

**Figure 2:**
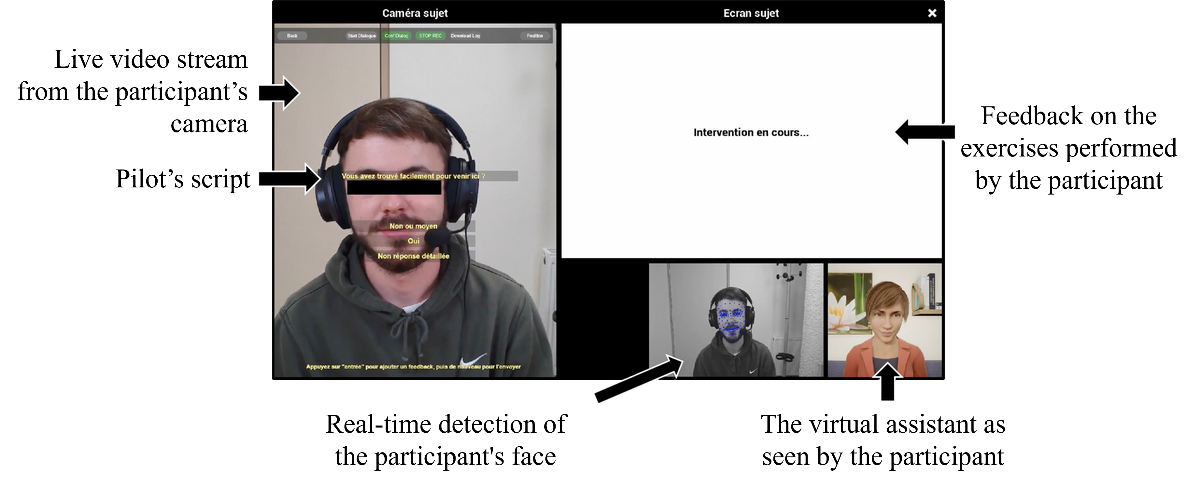
The pilot’s screen during a discussion between the virtual assistant and a participant (Atos software interface).

The speech of the virtual assistant was scripted and appeared on the screen of the pilot, who could thus read it and scroll it (refer to Figure 2 for a detailed view of the pilot screen). The main framework of the assistant’s speech was therefore identical from one participant to another; however, if necessary, the device allowed the pilot to intervene freely at any time during the session to help participants with questions or difficulties. In case of technical problems that could not be solved by the virtual assistant, the pilot informed the experimenter who could intervene.

The assistant’s speech was scripted to structure the session and provide the best support for the participant throughout the exercises. It was developed in line with the literature and the recommendations of experts working with older adults experiencing cognitive impairment, particularly with regard to the need for a reassuring, predictable environment and emotional support [40]. The assistant’s main roles are listed in Textbox 1.

**The main roles of the assistant.**

**Roles**

- Welcome the participant
- Explain the purpose of the session and exercises
- Explain how to complete the exercises
- Help participants with technical difficulties (eg, explain how to launch or perform an exercise and re-explain if necessary)
- Provide feedback on exercise performance
- Provide advice on how to improve performance
- Provide regular emotional support (eg, encourage participants, inquire about their emotional state, and suggest breaks if necessary)
- Provide information on cognitive functions and cognitive training in general
- Create a bond with the participant to create a comfortable environment and increase motivation (eg, make small talk and ask more personal questions)
- Handle any situation that may arise during a computerized cognitive training session (eg, need for a break, loss of motivation, or distraction)

Thus, this study has thus enabled us to test this script to perfect it and integrate it into the dialogue manager with an event-controlled finite state automaton that will be used for the final CCT software.

### Procedure

The participants were invited to the Université Lumière Lyon 2 (Bron, France) to perform a single session of CCT accompanied by a virtual assistant. The complete experiment lasted between 2 and 4 hours, depending on the participants. The average duration of the CCT session, including interactions with the virtual assistant, was around 1 hour and 15 minutes. After completing the consent form, the participants answered all the questionnaires assessing individual characteristics with the assistance of the experimenter. A break was suggested at the end of this first part, and participants were informed that they could take a break whenever they needed. Next, participants were seated in front of the computer and provided with headphones to perform the CCT with the virtual assistant. For this second part of the experiment, the experimenter left the room and let the participants attend the session alone.

The virtual assistant welcomed the participants and tried to get to know them, asking for some official information (name and age) and making some conversation about more personal topics, such as their job and hobbies. This first discussion was scripted in such a way as to make the participants feel comfortable and get them used to interacting with the assistant. The assistant then explained the interest of CCT in training cognitive functions and presented the course of the session, regularly asking questions to the participants.

Before each exercise, the virtual assistant gave the instructions and explained in an interactive way which cognitive functions were going to be trained. Then, the assistant disappeared for the duration of the exercise but could reappear to intervene if the participant had difficulty completing the exercise. After each exercise, the virtual assistant asked the participants how it went and gave them feedback on their performance, sometimes tips for improvement, and encouragement for the next exercise. Each exercise was performed twice, with the level of difficulty adjusted the second time based on the performance the first time. After the last exercise, the virtual assistant asked the participants how it went, whether they enjoyed the session, and which exercises they liked best and why. The assistant then thanked the participants before ending the CCT.

In the last part of the experiment, the participants answered the questionnaire evaluating the virtual assistant and the session with the help of the experimenter. Finally, they were informed about the Wizard of Oz device and were invited to meet the human pilot.

### Data Analysis

Data were analyzed using R (version 1.4; The R Foundation). The package *BayesFactor* (version 0.9.12-4.4) [55] was used to extract Bayes factors. Priors were set to default with an ultrawide scale [56]. The 95% credible interval (CI), representing the 95% highest density interval, was computed from posterior distribution using the package *bayestestR* (version 0.9.0) [57].

Analyses were performed using the Bayesian framework because it is more informative than the frequentist framework [58,59]. Indeed, rather than providing binary rejection information as the *P* value does, the Bayes factor (BF_10_) provides a level of evidence in favor of the alternative hypothesis against the null hypothesis. According to Kass and Raftery [60], BF_10_ can be interpreted as follows: BF_10_≥3 highlights moderate evidence, BF_10_≥10 highlights strong evidence, and BF_10_≥100 highlights decisive evidence.

We first tested whether the evaluation of the virtual assistant differed with age by comparing age groups with 1-tailed Bayesian *t* tests on each of the 7 scores of the assistant evaluation (ie, appreciation, comprehension, engagement, desire to give up, fatigue level, familiarity, and sense of humor). Then, for each age group separately, we performed Bayesian correlation analyses to investigate the relationship between the virtual assistant’s evaluation and individual characteristics (ie, psycho-affective functioning, personality, personal motivation, habits, and cognitive functioning). The groups were analyzed separately to highlight the specific profile of each population. Bayesian Pearson correlation coefficients and the corresponding BF_10_ were computed between the scores obtained on the questionnaires measuring participants’ characteristics and the 7 scores evaluating the virtual assistant. Descriptive data on participants’ responses to all questionnaires were also computed.

## Results

### Evaluation of the Virtual Assistant and Group Comparison

The mean ratings given by young and older participants to the virtual assistant are presented by dimension in Table 4. Results from Bayesian *t* tests suggested that young adults and older adults rated the assistant differently on all measures. Strong evidence was provided for the presence of a difference between age groups in the appreciation of the assistant (Cohen *d*=–0.32, 95% CI –0.70 to 0.05, BF_10_=23.00), comprehension of the exercises (Cohen *d*=–0.31, 95% CI –0.68 to 0.06, BF_10_>18.82), and desire to give up training (Cohen *d*=0.25, 95% CI –0.12 to 0.62, BF_10_=10.18). There was moderate evidence of an age-related difference in engagement (Cohen *d*=–0.19, 95% CI –0.55 to –0.18, BF_10_=5.70), as well as in ratings of familiarity (Cohen *d*=0.20, 95% CI –0.17 to 0.56, BF_10_=5.79) and sense of humor (Cohen *d*=0.17, 95% CI –0.19 to 0.54, BF_10_=4.69) of the assistant. Finally, decisive evidence was provided for the presence of a difference between groups in the level of fatigue reported at the end of the training (Cohen *d*=0.69, 95% CI 0.30-1.09, BF_10_>1000).

**Table 4:** Rating results for the virtual assistant evaluation questionnaire by dimension and age group.

Dimension	Older adults, mean (SD; range)	Young adults, mean (SD; range)	All, mean (SD)
Appreciation	7.88 (1.46; 3.57-9.80)	7.35 (1.43; 3-9.57)	7.62 (1.46)
Engagement	7.89 (1.25; 3.70-9.80)	7.62 (1.23; 4-9.9)	7.75 (1.24)
Comprehension	8.13 (1.37; 3.70-10)	7.67 (1.30; 4.25-9.80)	7.90 (1.35)
Desire to give up	1.17 (1.64; 0.20-8.30)	1.56 (1.75; 0.10-7.10)	1.36 (1.70)
Fatigue level	3.45 (2.45; 0.20-8.30)	5.19 (2.31; 0.40-9.30)	4.30 (2.53)
Familiarity	5.75 (1.25; 3.70-9.30)	6.02 (1.44; 3.50-8.50)	5.89 (1.35)
Sense of humor	6.71 (1.74; 3.90-9.80)	7.00 (1.59; 3.30-9.80)	6.85 (1.67)

### Correlational Analyses Between Individual Characteristics and Virtual Assistant Evaluation

#### Psycho-Affective Functioning

In young adults, analyses revealed moderate evidence for negative associations between the desire to give up and the following: arousal (*r*=–0.35, 95% CI –0.56 to –0.1, BF_10_=10.36), intrinsic relevance (*r*=–0.30, 95% CI –0.53 to –0.09, BF_10_=4.43), and goal conduciveness (*r*=–0.32, 95% CI –0.57 to –0.12, BF_10_=7.65). We also observed moderate evidence for the presence of a positive association between goal conduciveness and the overall appreciation of the virtual assistant (*r*=0.32, 95% CI 0.07-0.53, BF_10_=8.35). No evidence was provided for other correlations.

In older adults, moderate evidence was observed for a negative association between fatigue level and intrinsic relevance (*r*=–0.28, 95% CI –0.50 to 0, BF_10_=3.20) and for a positive association between fatigue level and state anxiety (*r*=0.28, 95% CI 0.04-0.53, BF_10_=3.84). No evidence was provided for other correlations. Participants’ scores on questionnaires assessing psycho-affective functioning are presented in Table 5.

**Table 5:** Rating results for the psycho-affective measures by questionnaire and age group.

Questionnaire and score		Young adults, mean (SD; range)	Older adults, mean (SD; range)
**Modified PANAS^a^**			
	Positive affect score	4.63 (0.92; 2.61-6.50)	4.58 (0.87; 2.28-6.17)
	Negative affect score	3.08 (0.82; 1.71-5.05)	2.11 (0.61; 1.14-3.90)
**Modified SAM^b^**			
	Arousal	5.46 (1.81; 1-9)	7.98 (1.23; 5-9)
	Intrinsic relevance	6.94 (1.16; 5-9)	8.00 (1.10; 5-9)
	Goal conduciveness	7.02 (1.20; 3-9)	7.81 (1.27; 5-9)
	Controllability	5.87 (1.77; 2-9)	6.73 (1.21; 3-9)
	Novelty	6.90 (2.52; 1-9)	7.27 (2.22; 1-9)
**BMIS^c^**			
	Pleasant-unpleasant	3.13 (0.36; 2.3-7.5)	3.55 (0.25; 2.94-4)
	Arousal-calm	2.22 (0.33; 1.25-3)	2.41 (0.31; 1.58-3.08)
**STAI-Y^d^**			
	State anxiety	1.52 (0.40; 1-3.15)	1.30 (0.24; 1-2.1)
	Trait anxiety	2.15 (0.53; 1.3-3.4)	1.75 (0.41; 1.05-2.75)

^a^PANAS: Positive and Negative Affect Schedule.

^b^SAM: Self-Assessment Manikin.

^c^BMIS: Brief Mood Introspection Scale.

^d^STAI-Y: State-Trait Anxiety Inventory.

#### Personality

The Bayes factor showed no evidence in favor of the presence of correlations between personality scores and the assistant’s evaluation in either young or older adults (refer to Table 6 for Ten-Item Personality Inventory scores).

**Table 6:** Ten-Item Personality Inventory (TIPI) scores by age group.

TIPI scores	Young adults, mean (SD; range)	Older adults, mean (SD; range)
Extraversion	4.13 (1.41; 1.5-7)	3.94 (1.21; 1-7)
Agreeableness	5.17 (0.96; 3.5-7)	5.38 (0.83; 3.5-7)
Conscientiousness	5.17 (1.19; 1.5-7)	5.85 (0.95; 3.5-7)
Emotional stability	3.85 (1.34; 2-6.5)	4.94 (1.14; 2-7)
Openness to experience	5.25 (1.06; 2-7)	5.18 (0.98; 2.5-7)

#### Personal Motivations

In young adults, the Bayes factor showed no evidence of correlations between personal motivation scores and the assistant’s evaluation. In older adults, results revealed moderate evidence of a negative correlation between intrinsic motivation toward knowledge and fatigue level (*r*=–0.26, 95% CI –0.52 to –0.06, BF_10_=3.02; refer to Table 7 for a description of Global Motivation Scale-28 scores).

**Table 7:** Global Motivation Scale (GMS)-28 scores by age group.

GMS-28 scores	Young adults, mean (SD; range)	Older adults, mean (SD; range)
Motivation toward knowledge	5.46 (1.21; 1.75-7)	6.00 (0.78; 4.25-7)
Motivation toward accomplishment	5.08 (1.44; 1.25-7)	4.82 (1.28; 2.25-7)
Motivation toward stimulation	5.20 (0.94; 2.75-7)	5.42 (0.99; 2.75-7)
Introjected motivation	4.25 (1.23; 1.75-6.75)	3.31 (1.25; 1-5.75)
Identified motivation	5.17 (1.05; 2.25-7)	4.13 (1.94; 5-6.75)
External motivation	3.94 (1.38; 1.25-6.75)	2.81 (1.54; 3.65-5.25)
Amotivation	2.76 (1.11; 1-5.75)	2.62 (1.19; 1-6)

#### Personal Habits

In young adults, analyses provided moderate evidence for a negative correlation between fatigue level and sport activity habit (*r*=–0.27, 95% CI –0.49 to –0.03, BF_10_=3.28). No other correlations were observed.

In older adults, moderate evidence was observed for a positive relationship between exercise engagement and familiarity with cognitive training exercises (*r*=0.27, 95% CI 0.01-0.53, BF_10_=3.33), as well as between the desire to give up and the habit of playing board games (*r*=0.28, 95% CI 0.04-0.50, BF_10_=3.67). No other correlations were observed. Descriptive statistics of participants’ responses to the questionnaire on personal habits and cognitive abilities are provided in Table 8.

**Table 8:** Rating results for the personal habits and cognitive abilities questionnaire by item and age group.

Personal habits and cognitive abilities			Young adults, mean (SD; range)		Older adults, mean (SD; range)
**Familiarity with...**					
	Computers	3.75 (0.74; 2-5)		3.42 (0.67; 2-5)	
	Cognitive exercises	2.53 (0.88; 1-4)		2.98 (1.04; 1-5)	
	Cognitive training	1.94 (0.75; 1-4)		2.48 (1.04; 1-4)	
**Cognitive abilities**					
	Memory difficulty	3.56 (0.67; 2-5)		3.65 (0.62; 3-5)	
	Attentional difficulty	3.52 (0.92; 2-5)		3.88 (0.83; 2-5)	
**Frequency of activities**					
	Playing a musical instrument	1.98 (1.13; 1-5)		1.35 (0.86; 1-5)	
	Playing board games	3.23 (1.02; 1-5)		3.15 (0.98; 1-5)	
	Playing games such as chess or crossword puzzles	2.17 (1.00; 1-5)		3.06 (1.32; 1-5)	
	Sports and exercise	3.54 (1.13; 1-5)		4.19 (0.66; 2-5)	
	Meditation and relaxation	2.04 (1.10; 1-5)		2.31 (1.26; 1-5)	

#### Cognitive Functioning (Older Adults Only)

Moderate evidence was observed for a positive correlation between overall cognitive functioning (as measured by MMSE total score) and exercise engagement (*r*=0.31, 95% CI 0.09-0.55, BF_10_=6.28). No evidence was provided for other correlations. Descriptive statistics of older adults’ performance on the questionnaires measuring cognitive functioning are presented in Table 9.

**Table 9:** Older adults’ scores on questionnaires measuring cognitive functioning.

Questionnaire and score		Values, mean (SD; range)
**MMSE^a^**		
	Total score	28.96 (1.24; 25-30)
**TMT^b^**		
	Execution time	82.62 (32.22; 47.5-208)
	Number of errors	0.30 (0.69; 0-4)
**FAB^c^**		
	Total score	17.10 (1.42; 13-18)
**5WT^d^**		
	Total score	9.90 (0.45; 7-10)

^a^MMSE: Mini-Mental State Examination.

^b^TMT: Trail Making Test.

^c^FAB: Frontal Assessment Battery.

^d^5WT: 5 Words Test.

## Discussion

### Principal Findings

In this study, we explored the interest of adding a virtual assistant during CCT, with the objective of improving patients’ adherence to cognitive training programs performed autonomously at home. To this end, we recruited young and older adults to complete and evaluate a CCT session conducted by a virtual assistant and explored the relationship between their evaluation and various individual factors (ie, age, psycho-affective functioning, personality, personal motivations, and cognitive skills). Overall, the results suggested that a virtual assistant would be appreciated and useful during CCT in both age groups. Certain characteristics of users, especially their current affective state, would be related to their evaluation of the session.

The high appreciation scores showed that both young and older adults felt well accompanied by the virtual assistant during CCT. The virtual assistant appeared to have had a beneficial impact on exercise comprehension and motivation, as suggested by the strong engagement and very low desire to give up reported by both groups. The level of fatigue declared at the end of the session was fairly mild and can be partly explained by the novelty of the device and the experimental context. As for the assistant’s personality, both groups would have preferred it to be more familiar and humorous; therefore, these parameters should be considered when developing such an assistant. A recent review of the literature showed that other parameters regarding conversational style should also be considered [61]. For example, virtual health assistants exhibiting nonverbal relational behaviors and self-disclosure were associated with a better user experience. In addition, these same authors stressed the importance of a realistic rendering of the assistant’s appearance, evoking a medical context. However, there may be cultural differences in design preferences for virtual assistants. One study showed, for example, a preference for strong realism among older participants from the Netherlands, while Swiss participants preferred a cartoon-like appearance [34]. One solution could be to offer avatar customization options in this kind of software. Further research on the optimal design of virtual assistants is nevertheless necessary.

Moreover, Bayesian analyses brought evidence for differences between age groups on all dimensions assessed. Older adults appreciated the virtual assistant slightly more than young adults and reported higher engagement and better comprehension of the exercises. They reported less desire to give up and less fatigue at the end of training than their younger counterparts. The main explanation for these differences is certainly that this version of the device was specifically conceived for older adults with or without cognitive impairments, considering their preferences and needs, which may differ from those of young adults [32-35,40,41]. Young adults may also have felt less concerned by cognitive training; adaptations will be necessary to propose the device to a younger public experiencing cognitive disorders. For example, analyses showed that familiarity and sense of humor were more important for young than for older adults, suggesting that the assistant’s personality should be adapted according to the target audience. In addition, there is some evidence that young adults may prefer to interact with less realistic, nonhuman virtual assistants (eg, zoomorphic or machine-like assistants), unlike older adults [32].

Because older adults’ responses tended to amplify the beneficial aspects of the virtual assistant during CCT and minimize the negative effects, such as the desire to give up or fatigue, it is also possible that a social desirability bias was at work in older adults. This bias refers to people’s tendency to present themselves in an overly positive manner in self-reports [62], and it has been shown to increase with age, especially when it comes to reports of well-being, depressive symptoms, and mood [62,63]. The differences observed between age groups were nevertheless quite small on all dimensions measured, except for fatigue, where older adults reported a much lower level of fatigue than young adults. Because fatigue may be a more direct reflection of health and self-image than the other measures, which may both be negatively impacted by aging, it seems possible that the social desirability bias would be particularly visible in this dimension.

Bayesian correlations allowed us to identify interesting associations between some individual characteristics and the evaluation of the virtual assistant. Psycho-affective functioning, especially affective state at the time of the session, appeared to play an important role in both age groups. In young adults, the results showed that 3 parameters of current affective state would be moderately associated with the desire to give up the session: goal conduciveness, arousal, and intrinsic relevance. As goal conduciveness (ie, the consistency of the situation with current concerns) increased, the desire to give up decreased and the appreciation of the virtual assistant increased, suggesting that goal conduciveness would be particularly associated with young adults’ motivation during CCT. In addition, the higher the arousal (ie, state of alertness) and intrinsic relevance (ie, level of pleasure) at the time of the session, the lesser the desire young adults had to give up the session.

The results obtained in older adults also highlighted the importance of current affective state (ie, intrinsic relevance and anxiety state) during CCT but in relation to the level of fatigue reported at the end of the session. Indeed, older adults’ fatigue increased with anxiety state and decreased as intrinsic relevance increased. To minimize fatigue during CCT, help from the virtual assistant to manage anxiety could therefore be beneficial. In both age groups, no evidence was provided for correlations between the assistant’s evaluation and global affective experience in everyday life (modified Positive and Negative Affect Schedule), anxiety trait (State-Trait Anxiety Inventory), and some other measures of current affective state (Brief Mood Introspection Scale scores, controllability, and novelty). We did not observe any relationships between psycho-affective functioning and participants’ engagement in and comprehension of the exercises.

Nevertheless, our data overall suggest that different dimensions of emotional state, such as arousal, goal conduciveness, intrinsic relevance, and anxiety, are likely to modulate participants’ appreciation of the CCT and their motivation (ie, desire to give up and fatigue), which could eventually impact adherence to the training program. The ability to detect and react to emotional states would therefore be a particularly useful feature for a virtual assistant in CCT, which would contribute to maintaining or even improving motivation [42]. This proposition is consistent with the available literature, suggesting that virtual health assistants who demonstrate empathy are associated with a more positive user experience [61] and may increase adherence by giving the impression of being understood [40]. When developing an empathetic virtual assistant, for example, the detection of anxiety in the user’s facial expression or voice could lead the assistant to question them about the cause of their anxiety, to reassure them, to propose a break, or to adapt the difficulty level of the exercises.

Our analyses did not provide evidence for correlations between users’ personality traits (based on the Big Five personality traits) and the evaluation of the assistant in any age group. Moreover, no relationship was observed in young adults between their personal motivations and the assistant’s evaluation, whereas older adults presented a decrease in the level of fatigue as intrinsic motivation toward knowledge increased. We also observed some correlations with personal habits (eg, sports activity, familiarity with cognitive training exercises, or playing board games) in both age groups. In young adults, high sports activity was associated with low fatigue at the end of CCT. In older adults, we observed that (1) the more they were used to cognitive training exercises, the more engaged they felt during CCT, and (2) the more they were used to playing board games, the more they desired to give up the session. Further investigations are necessary to clarify these results.

Interestingly, we did not observe any correlation between computer familiarity and session evaluation. However, the CCT in our study was led by a human pilot who was able to provide optimal support by reacting appropriately to any situation. For home-based CCT, without human assistance, one can expect that computer familiarity will be a determining factor in handling the CCT software. A virtual assistant would be a key element in ensuring the success of cognitive training by directly answering users’ questions and helping them solve their difficulties, especially among those who are not familiar with computers. However, as older adults have expressed their need for personalized help in acquiring knowledge of new technologies [64], minimal training in using the CCT software will remain necessary and can be provided by health professionals.

Analyses also revealed that exercise engagement positively correlated with overall cognitive functioning (assessed by MMSE total score) in older adults. This result means that older adults with low cognitive functioning would be likely to be less engaged in completing the exercises. This is a delicate point because CCT with or without an assistant is aimed particularly at people with, or at risk of, cognitive disorders. Furthermore, cognitive training is typically prescribed at an average of 1 to 2 sessions per week over a minimum of 8 weeks to several months to have a beneficial effect [14,15,65,66], so the repetitiveness of the sessions is likely to cause a drop in motivation. In line with the propositions made earlier, extreme attention should then be paid to the management of motivation and reassurance of patients when developing a virtual assistant to accompany CCT. In this regard, this exploratory study has 2 major limitations. First, we have not yet collected the opinions of patients with MCI on CCT with a virtual assistant. It is indeed possible that patients with cognitive disorders may evaluate the virtual assistant differently from healthy people. Nevertheless, we did anticipate possible discrepancies by considering the particularities of patients with cognitive impairment when developing the virtual assistant. The assistant’s script was notably conceived in line with the recommendations of experts working with older adults with cognitive disorders [40]. The second limitation of our study is that it provides no information on the effectiveness of our device in the training of cognitive functions, compared to CCT without a virtual assistant. On the basis of the data collected in this first study, including the videos of the human pilot and participants, we are currently developing the first version of our future autonomous virtual assistant [42]. The videos of the human pilot will be used to develop the facial expressions and voice of the virtual assistant, and the participants’ videos will be used to train our artificial intelligence to autonomously detect users’ facial expressions, particularly those expressing emotions and fatigue, so that the virtual assistant can react appropriately. The next step in our work will be to test this autonomous agent with patients with MCI in a longitudinal approach to evaluate the benefits of cognitive training accompanied by a virtual assistant in the long term.

In this context, the last topic that we wanted to address concerns the technology that will underpin our virtual assistant and virtual assistants in general. In this study, interactions between the assistant and user were scripted: this enabled us to test a series of adapted dialogues, with the aim of using them later to develop a dialogue manager with an event-controlled finite state automaton. While we were conducting this study and writing this paper, large language models such as ChatGPT were undergoing significant development. However, dialogue managers with a finite number of possible interactions have certain advantages, especially for patients with cognitive disorders. First, such a device allows us to master and certify all verbal content, thus providing a stable and rather predictable environment for those patients who may have comprehension difficulties. Although popular generative models such as ChatGPT have not been technically disclosed, it is known that human knowledge is used by reinforcement learning to avoid systems providing misleading information, particularly on at-risk topics such as health or religion. However, these limitations are not clearly defined and vary according to model updates, so the risk of leading the user to inappropriate actions or behaviors due to misinterpretation of the model is far from negligible. People with cognitive disorders need a safe environment in which to interact with a virtual assistant, which requires total control over the possible responses given by the technology. Second, we avoid confidentiality and ethical issues by not basing our virtual assistant on this technology. Indeed, the European Union Artificial Intelligence Act [67] will specifically ban artificial intelligence systems with unacceptable risks that include cognitive behavioral manipulation of specific vulnerable individuals or groups. Finally, we have more control over processing issues such as response time using cost-effective and lightweight processing with no graphics processing units. However, we do use large language models to enhance the capacity of the virtual assistant to detect the user’s intention and emotion. The dialogue editor also uses the ChatGPT application programming interface to facilitate the work of scriptwriters, notably by generating paraphrases to avoid too repetitive interventions. All scripts are examined and revised by human scriptwriters.

### Conclusions

The recent COVID-19 pandemic has emphasized the urgency of developing digital health technologies, as they are a useful tool for remote monitoring and can help ensure continuity of patient follow-up [68]. In our aging population, the number of individuals with cognitive impairment, MCI, and dementia is expanding, and CCT is a key solution for patients to continue their training at home. Because the lack of social interactions may contribute to the lower effectiveness of home-based CCT [9], the addition of a virtual assistant in CCT would allow for a more stimulating accompaniment with social interactions that would compensate for the absence of a therapist and reduce the feelings of loneliness often reported by older adults [69]. This study has shown that such a virtual assistant would be appreciated by young and older adults and could have a beneficial influence on users’ motivation, provided that it can handle different situations and, in particular, take into account their emotional state. Following this exploratory study, the next step will be to evaluate our solution with patients with MCI and test its effectiveness in long-term cognitive training.

## Abbreviations

BF10: Bayes factor
CCT: computerized cognitive training
CI: credible interval
FAB: Frontal Assessment Battery
MCI: mild cognitive impairment
MMSE: Mini-Mental State Examination
THERADIA: Thérapies Digitales Augmentées par l’Intelligence Artificielle
TMT: Trail Making Test
